# ABC-GWAS: Functional Annotation of Estrogen Receptor-Positive Breast Cancer Genetic Variants

**DOI:** 10.3389/fgene.2020.00730

**Published:** 2020-07-20

**Authors:** Mohith Manjunath, Yi Zhang, Shilu Zhang, Sushmita Roy, Pablo Perez-Pinera, Jun S. Song

**Affiliations:** ^1^Department of Physics, University of Illinois at Urbana-Champaign, Urbana, IL, United States; ^2^Carl R. Woese Institute for Genomic Biology, University of Illinois at Urbana-Champaign, Urbana, IL, United States; ^3^Department of Bioengineering, University of Illinois at Urbana-Champaign, Urbana, IL, United States; ^4^Wisconsin Institute for Discovery, University of Wisconsin–Madison, Madison, WI, United States; ^5^Department of Biostatistics and Medical Informatics, University of Wisconsin–Madison, Madison, WI, United States; ^6^The Carle Illinois College of Medicine, Champaign, IL, United States; ^7^Cancer Center at Illinois, University of Illinois at Urbana-Champaign, Urbana, IL, United States

**Keywords:** GWAS, breast cancer, functional characterization, variant annotation, web resource

## Abstract

Over the past decade, hundreds of genome-wide association studies (GWAS) have implicated genetic variants in various diseases, including cancer. However, only a few of these variants have been functionally characterized to date, mainly because the majority of the variants reside in non-coding regions of the human genome with unknown function. A comprehensive functional annotation of the candidate variants is thus necessary to fill the gap between the correlative findings of GWAS and the development of therapeutic strategies. By integrating large-scale multi-omics datasets such as the Cancer Genome Atlas (TCGA) and the Encyclopedia of DNA Elements (ENCODE), we performed multivariate linear regression analysis of expression quantitative trait loci, sequence permutation test of transcription factor binding perturbation, and modeling of three-dimensional chromatin interactions to analyze the potential molecular functions of 2,813 single nucleotide variants in 93 genomic loci associated with estrogen receptor-positive breast cancer. To facilitate rapid progress in functional genomics of breast cancer, we have created “Analysis of Breast Cancer GWAS” (ABC-GWAS), an interactive database of functional annotation of estrogen receptor-positive breast cancer GWAS variants. Our resource includes expression quantitative trait loci, long-range chromatin interaction predictions, and transcription factor binding motif analyses to prioritize putative target genes, causal variants, and transcription factors. An embedded genome browser also facilitates convenient visualization of the GWAS loci in genomic and epigenomic context. ABC-GWAS provides an interactive visual summary of comprehensive functional characterization of estrogen receptor-positive breast cancer variants. The web resource will be useful to both computational and experimental biologists who wish to generate and test their hypotheses regarding the genetic susceptibility, etiology, and carcinogenesis of breast cancer. ABC-GWAS can also be used as a user-friendly educational resource for teaching functional genomics. ABC-GWAS is available at http://education.knoweng.org/abc-gwas/.

## Introduction

Genome-wide association studies (GWAS) have implicated thousands of genetic variants in various complex traits, including diseases (MacArthur et al., 2017). However, only a few studies to date have been successful in characterizing the underlying molecular mechanisms that govern how genetic variations affect molecular interactions (Musunuru et al., 2010; Cowper-Sal lari et al., 2012; Bauer et al., 2013; Huang et al., 2014; Smemo et al., 2014; Gallagher et al., 2017; Zhang et al., 2018b). Studying the molecular function of a typical GWAS locus presents several key challenges (Gallagher and Chen-Plotkin, 2018). First, most of the variants found through GWAS are located in non-coding regions of the human genome; as a result, the precise link between a non-coding variant and some target protein’s function is not immediately clear. Second, GWAS variants may indirectly correlate with a phenotype through a complex gene regulatory network involving multiple target genes, unknown causal variants, and transcription factors (TFs). For example, a reported GWAS variant may simply be genetically linked to another proximal variant that itself directly perturbs the binding affinity of a TF and changes the expression of a distal target oncogene or tumor suppressor forming a chromatin loop with the causal variant. In such cases, there is the additional complexity of having to dissect how different components of a gene regulatory network are altered and function together to modulate a trait. Finally, functional characterization of GWAS loci must be carried out in the right cell type representing the phenotype in question; however, one often lacks a complete set of data in genomic, epigenomic, and transcriptomic contexts in the cell type of interest or even faces a difficulty in determining the right cell type. Therefore, there is an urgent need for comprehensive and easily accessible resources that integrate information from heterogeneous large-scale datasets to facilitate rapid functional characterization of GWAS findings and ultimately contribute toward the development of therapeutic preventions and interventions.

Building on the public catalog of GWAS variants (MacArthur et al., 2017), there are currently a few databases providing functional annotation of disease variants. The GRASP database annotates GWAS results by summarizing millions of single nucleotide variant-phenotype associations from 1,390 GWAS studies through correlations such as expression quantitative trait loci (eQTLs), metabolite QTLs, and methylation QTLs (Leslie et al., 2014). Similarly, GWASdb curates trait-associated single nucleotide polymorphisms (SNPs) with detailed functional annotations including eQTL and disease ontology terms (Li et al., 2016). Phenoscanner is a curated database containing variant-phenotype associations of several types such as disease, methylation, gene expression, and protein levels (Staley et al., 2016). More recently, Qtlizer provides associations of variants with gene expression levels and protein abundance using published QTLs (Munz et al., 2019). In the context of cancer, PancanQTL provides a comprehensive list of cis- and trans-eQTLs, including GWAS-related eQTLs, in 33 cancer types (Gong et al., 2018). These web resources have specific advantages, such as having a detailed annotation of GWAS SNPs and/or a list of potential target genes found through eQTL analysis. However, these resources do not perform an in-depth integrative analysis of a specific cancer type using state-of-the-art information about cell type-specific epigenetic landscape, chromatin contact interactions, and TF binding affinity, required for a complete functional characterization of GWAS loci.

Most studies investigating breast cancer GWAS variants have so far focused only on eQTL analysis to find genes correlated with a variant genotype, while only few have pursued a systematic analysis of causal variants and target genes through chromatin structure and TFs (Cowper-Sal lari et al., 2012; French et al., 2013; Li et al., 2013; Ghoussaini et al., 2014, 2016; Darabi et al., 2015; Dunning et al., 2016; Michailidou et al., 2017; Zhang et al., 2018b; Zhang Y. et al., 2019). This paper presents ABC-GWAS, an interactive database containing our comprehensive analysis of 70 manually curated estrogen receptor-positive (ER+) breast cancer GWAS loci and 23 additional ER+ breast cancer loci from a recent fine mapping study (Fachal et al., 2020). The set of 70 loci was obtained from the literature on breast cancer GWAS (Turnbull et al., 2010; Michailidou et al., 2013, 2015). Utilizing large-scale multi-omics datasets such as the Cancer Genome Atlas (TCGA) and the Encyclopedia of DNA Elements (ENCODE) (ENCODE Project Consortium, 2012), our analysis pipeline includes eQTL analyses for identifying putative target genes, causal variant prioritization utilizing relevant epigenomic datasets, motif and expression correlation analyses for identifying putative TFs, and three-dimensional chromatin contact predictions for assessing long-distance enhancer-gene interactions. ABC-GWAS aggregates and organizes these results, not readily available in other existing databases, via a user-friendly web interface, making them easily accessible to researchers for additional analysis or experimental validation. It features an embedded genome browser that includes histone modification, chromatin interaction, and TF chromatin immunoprecipitation followed by sequencing (ChIP-seq) tracks for further exploration of the GWAS locus and linked non-coding variants of interest. ABC-GWAS also shows the average DNA copy number information in TCGA breast cancer samples at each GWAS locus. Our resource thus provides useful practical results and conceptual approaches to the functional genomics community in general and breast cancer researchers in particular.

## Materials and Methods

### TCGA Data and Genotype Imputation

The processed RNA-seq expression data in RSEM (RNA-Seq by Expectation-Maximization) units for 794 ER+ breast cancer patients were obtained from the TCGA Genomic Data Commons (GDC) Legacy Archive (Grossman et al., 2016). The germline genotypes of 788 patients in birdseed format for TCGA-BRCA (Breast Invasive Carcinoma) patients were also obtained from the TCGA Data Portal. The copy number segmentation data for 693 patients in hg19 coordinates were retrieved from the GDC Legacy Archive (Grossman et al., 2016). For genotype imputation of the raw genotypes in birdseed format, confidence score greater than 0.1 was used to mark the probed genotypes as missing, which was then imputed along with the non-probed SNPs. We used the Michigan Imputation Server for imputation (Das et al., 2016), choosing the Haplotype Reference Consortium (HRC) r1.1 2016 as a reference panel (Loh et al., 2016a), Eagle v2.3 for phasing (Loh et al., 2016b), and EUR population as the quality control option. Imputed genotypes were retained if the minor allele frequency (MAF) exceeded 0.005 and estimated imputation accuracy (R^2^) exceeded 0.4.

### Credible Causal Variants in 23 Additional GWAS Loci

We obtained the full list of credible causal variants (CCVs) from Fachal et al. (2020) and then selected the variants that are single-nucleotide variants, associated with ER+ breast cancer (column ERpos = 1), and have posterior probability of being causal greater than zero (column PP_ERpos > 0). We further removed SNPs that did not pass the quality control tests in the Michigan Imputation Server or for which genotypes could not be imputed confidently in the TCGA data. Finally, excluding 227 CCV SNPs already present in the list of 2,510 SNPs that were in high linkage disequilibrium (*r*^2^ > 0.8, 1000 Genomes Phase 3, EUR population) with the reported GWAS SNPs in the 70 manually curated regions yielded 303 CCVs with non-zero posterior probability of being causal in ER+ breast cancers. The 303 CCVs resided in 32 GWAS regions, and 23 of these regions differed from the 70 manually curated regions. ABC-GWAS thus contains the analysis of 530 CCVs out of the 1,238 CCVs reported for ER+ breast cancer.

### Genome Browser

The WashU EpiGenome Browser source code was obtained from their GitHub repository (Li et al., 2019; WashU, 2019). The browser uses hg19 coordinates. The JavaScript files from the source code were used to generate the tracks in the embedded browser of ABC-GWAS. The tracks included TF ChIP-seq peaks publicly available in ReMap 2018 database (Cheneby et al., 2018), ENCODE DNase-seq signals, and ESR1, GATA3, and FOXA1 ChIP-seq signals in MCF-7 and T-47D cell lines, POLR2A, CTCF, and ESR1 ChIA-PET interactions, and chromatin interaction predictions in MCF-7 cell line. CTCF is known to play an important role in defining the activity of ESR1 in ER+ breast cancer (Carroll et al., 2006; Chan and Song, 2008). The above datasets were downloaded from the corresponding sources and integrated into our server (Supplementary Table 1).

### Chromatin Interaction Predictions

To predict SNP-associated interactions, we applied HiC-Reg (Zhang S. et al., 2019), a tool for predicting Hi-C contact counts between pairs of genomic loci from their one-dimensional regulatory signals such as histone modification data, TF ChIP-seq, and chromatin accessibility. We obtained ChIP-seq datasets for 10 histone marks and TFs, and DNase-seq datasets in five cell lines from ENCODE (Supplementary Material). HiC-Reg can be trained using cell-line-specific datasets for a cell line with available high-resolution (5 kb) Hi-C data, e.g., the five human cell lines available from Rao et al. (2014). Once trained, HiC-Reg takes as input the genomic features associated with a pair of regions and predicts the chromatin contact count for that pair. We used the method to make predictions in the MCF-7 cell line by training eight different models at 5 kb resolution (Supplementary Material). To interpret our results, we averaged the predictions across eight models and displayed the resulting contact count profile associated with each SNP on ABC-GWAS.

### eQTL Analysis

To identify candidate target genes for each GWAS SNP, we scanned all genes within 4 Mb centered at the SNP by constructing a multivariate linear regression model with the expression level of each gene as the response variable and the genotype of the GWAS SNP and the copy number (CN) of the gene as predictors (Zhang et al., 2018b; Zhang Y. et al., 2019). The processed gene expression levels in RSEM units were transformed as *log*_2_⁡(*RSEM* + 1). The patients with ER+ breast cancer based on TCGA clinical information were retained for subsequent analysis. The genotypes of each GWAS SNP were encoded as the number of risk alleles based on the risk allele information from the NHGRI GWAS catalog (MacArthur et al., 2017). The tumor copy number segmentation values were transformed into gene copy number by taking gene length-weighted average and using *CN* = 2×2^{*segmentation*}^. We then performed multivariate linear regression and selected genes with mean RSEM larger than 1 and genotype *p-*value less than 0.05 as candidate target genes for each breast cancer GWAS SNP. On the website, a violin plot using plotly.js is displayed to show the distribution of a candidate target gene’s mRNA expression as a function of the GWAS SNP’s genotype status (Plotly, 2018).

### ENCODE Data

ChIP-seq files for 715 TFs and histone marks in 231 cell lines and tissues were obtained from the ENCODE website (Davis et al., 2018). The locations of the breast cancer risk variants along with the high LD SNPs were then intersected with the peaks of each TF or histone mark in every cell line using bedtools (Quinlan and Hall, 2010). A list of TFs, relevant cell lines, and distance of the SNP from peak center were then tabulated for display.

### Motif Analysis

Position weight matrices (PWM) for TFs were obtained from several public databases included in the MotifDb and motifbreakR packages on Bioconductor (Coetzee et al., 2015; Shannon, 2017). The public databases included Jaspar 2018 (Khan et al., 2018), HOCOMOCO (Kulakovskiy et al., 2018), hPDI (Xie et al., 2010), Jolma (Jolma et al., 2013), cisbp (Weirauch et al., 2014), UniPROBE (Hume et al., 2015), Swiss Regulon (Pachkov et al., 2013), HOMER (Heinz et al., 2010), ENCODE motifs (Kheradpour and Kellis, 2014), and FactorBook (Wang et al., 2012). TRANSFAC matrices were also added to the above list (Wingender, 2008). In the first step, the motifbreakR package was used to get possible motif disruptions by candidate SNPs with a *p*-value threshold of 10^–3^. We then used our previously developed random mutation model to test the significance of difference in motif scores for the two sequences carrying reference and alternative alleles (Zhang et al., 2018b). The motif disruptions that passed the permutation test *p*-value threshold of 0.05 were denoted as significant and subsequently included in the ABC-GWAS database.

### Correlation Analysis

The list of putative TFs from motif analysis was filtered by removing TFs whose log-transformed mean expression levels across TCGA ER+ breast cancer patients were less than 1 (mean *log*_2_⁡(*RSEM* + 1) < 1). For each putative target gene from eQTL analysis and TFs passing the expression cut-off threshold, we computed the Pearson correlation coefficient between the expression levels of the target gene and TF across TCGA ER+ breast cancer primary tumor samples, stratifying the patients into three genotype groups: homozygous-risk, heterozygous, and homozygous-alternative. We reasoned that for a good candidate TF, the correlation should be strongest in the homozygous genotype group preserving the TF motif and weakest in the homozygous genotype group disrupting the motif.

## Results

### Analysis Pipeline for Prioritization of Functional Candidates

**FIGURE 1:**
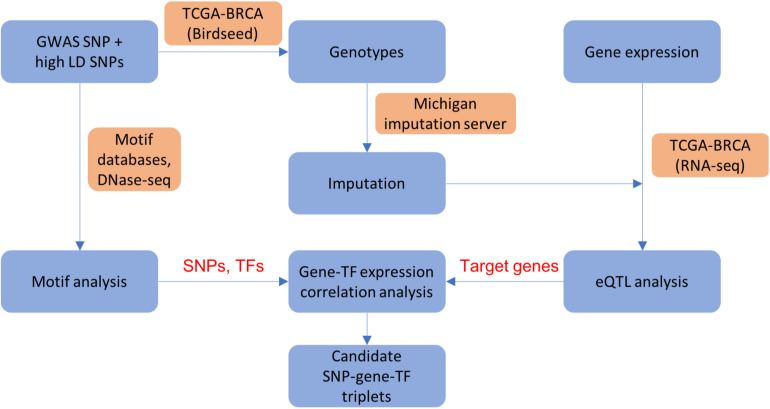
A flowchart showing integrative analysis pipeline used in ABC-GWAS. For each GWAS locus, we perform eQTL analysis, motif analysis, and gene-TF expression correlation analysis to obtain candidate (SNP, gene, TF) triplets. Blue boxes show analysis steps, orange boxes indicate data and/or resources used in the analysis, and text in red shows intermediate results.

We applied the analysis pipeline from our previous work (Zhang et al., 2018b), summarized in Figure 1, on a list of manually curated ER+ breast cancer GWAS variants and all SNPs in high linkage disequilibrium (LD) with the GWAS variants, as well as an additional 303 credible causal variants (CCVs) with non-zero posterior probability of being causal in ER+ breast cancers (Fachal et al., 2020; section “Materials and Methods”). The basic framework performs various genomic analyses outlined below to infer how a GWAS variant or a linked SNP changes the binding affinity of a TF in a regulatory region, which in turn alters the transcription of a target gene. In our analysis, linked SNPs residing in accessible open chromatin sites with activating histone modifications (H3K4me1 and H3K27ac) are prioritized as candidate causative SNPs. The genotypes and gene expression data were obtained from TCGA, where the non-probed SNPs’ genotypes were imputed using the Michigan imputation server (Das et al., 2016; section “Materials and Methods”). We gathered various heterogeneous datasets from high-throughput experimental techniques such as DNase I hypersensitive sites sequencing (DNase-seq) for prioritization of candidate causal variants, ChIP-seq for TF binding evidence, and chromatin interaction analysis with paired-end tag sequencing (ChIA-PET) and RNA-seq for target gene prioritization in breast cancer samples or cell lines (section “Materials and Methods”; Supplementary Table 1). In order to assess how a SNP may perturb a TF’s binding affinity and consequently modulate a target gene’s expression, we performed eQTL analysis, motif analysis, and TF vs. target gene expression correlation analysis to determine a list of candidate (SNP, target gene, TF) triplets (section “Materials and Methods”).

### ABC-GWAS User Interface

**FIGURE 2:**
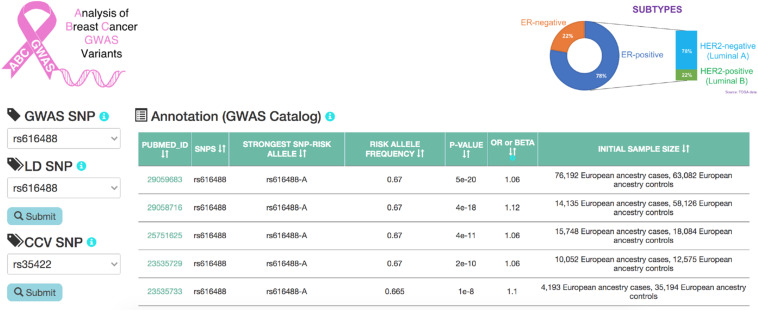
A snapshot of the homepage of ABC-GWAS. Selecting a GWAS SNP using the left-hand-side drop-down menu populates the table on the right with relevant GWAS publications. Upon selecting an LD SNP or a CCV and clicking on the “Submit” button, various tabs on the bottom containing analysis modules are loaded.

**FIGURE 3:**
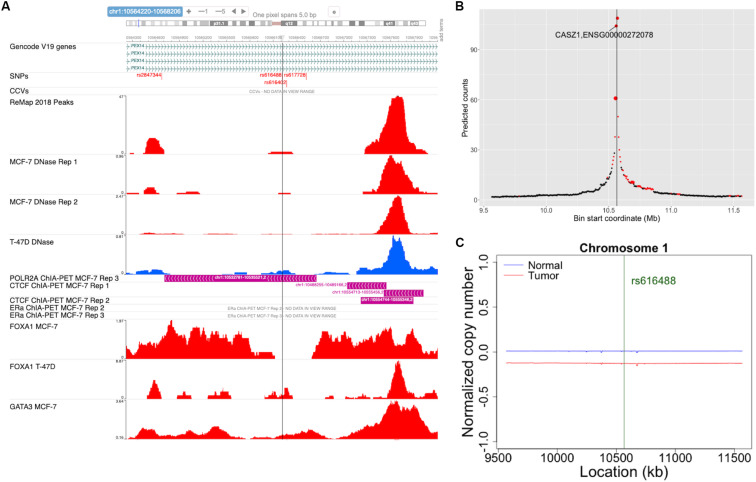
Snapshots of some of the ABC-GWAS analysis modules. **(A)** Embedded genome browser showing the queried LD SNP location (vertical line) and ChIP-seq tracks in MCF-7 and T-47D cell lines. The “SNPs” track shows the locations of the GWAS SNPs and high LD SNPs, while the “CCVs” track shows the locations of the credible causal variants and their GWAS lead SNPs. **(B)** The plot shows average predicted chromatin contact counts across several HiC-Reg models in MCF-7 as a function of genomic location centered at the queried LD SNP (vertical line). The predictions for which at least one model shows significance (*q* < 0.05) is filled in red. The size of the markers is proportional to the number of models showing significance. **(C)** Normalized DNA copy number for normal (blue) and tumor (red) samples from TCGA in a 2 Mb window centered at the GWAS SNP location.

ABC-GWAS is divided into several modules for interactive data exploration. In the query module, the user first selects a GWAS SNP of interest from the list of 70 SNPs which represent the best reported variants in the manually curated implicated loci, after which a list of high LD (*r*^2^ > 0.8, 1000 Genomes Phase 3, EUR population) SNPs of the queried GWAS SNP is populated (Figure 2). A table containing the list of GWAS studies implicating the selected SNP in breast cancer is shown on the right-hand side of the query module (Figure 2). Alternatively, the user may choose one of the additional 303 CCVs, not found in the list of all high LD SNPs. After submitting a high LD or CCV SNP as the query variant, all the analysis tabs below the query module get updated. The first tab contains an embedded genome browser showing ChIP-seq, DNase-seq, and ChIA-PET sequencing tracks around the queried SNP locus (Figure 3A; section “Materials and Methods”). The second tab displays predicted chromatin interactions in the MCF-7 breast cancer cell line, showing significant interactions between the queried LD SNP location and nearby gene promoters (Figure 3B; section “Materials and Methods”, and Supplementary Material); this track is not available for the 303 CCVs. The third tab consists of two modules. One module shows the average DNA copy number around the queried GWAS SNP location using the TCGA copy number segmentation data for normal and tumor samples (Figure 3C; section “Materials and Methods”). The other module checks whether the queried SNP is a CCV (Fachal et al., 2020); when available, a list of likely target genes of the queried SNP obtained from the same study is also displayed. The fourth tab summarizes our eQTL analysis results for the selected GWAS SNP or CCV using the genotypes and RNA-seq data from TCGA breast cancer samples (section “Materials and Methods”). A table containing significant eQTL results and a violin plot of the target gene’s expression stratified into genotype groups are displayed. The fifth tab shows a table of all ENCODE ChIP-seq peaks that intersect the queried SNP (section “Materials and Methods”). The peaks are categorized based on whether the experiment is for a TF or histone modification. The results can also be filtered to show peaks occurring only in breast tissue or breast cancer-related cell lines. The last tab contains two modules showing putative TFs, the binding activities of which are predicted to be affected by the given SNP, as assessed by motif analysis (section “Materials and Methods”) and expression correlation analysis (section “Materials and Methods”). A motif logo with the nucleotide perturbed by the SNP is available for each of the putative TFs. In the “Expression correlation” tab, the putative TFs from motif analysis are further prioritized based on the expression correlation between each TF and eQTL target genes. Pearson correlation coefficients are displayed as a heatmap with the putative TFs along the rows and genotype groups along the columns.

### Case Study (Validated Result From the Literature): (rs4784227, *TOX3*, FOXA1)

**FIGURE 4:**
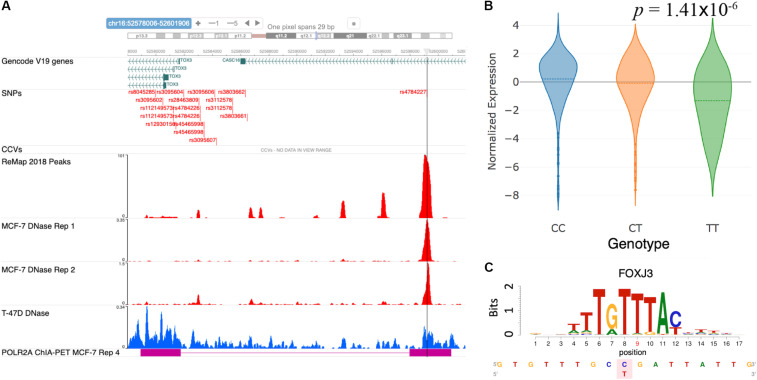
Case study of GWAS locus at *TOX3*: (rs4784227, *TOX3*, FOXA1). **(A)** Embedded genome browser showing the SNP rs4784227 within an open chromatin region (MCF-7 DNase tracks) and several TF peaks (“ReMap 2018 Peaks”). **(B)** eQTL plot showing significant correlation between *TOX3* expression and rs4784227 genotypes. **(C)** Predicted TF candidate FOXJ3 motif from Kheradpour and Kellis (2014).

Cowper-Sal lari et al. (2012) analyzed the functional mechanism of the GWAS SNP rs4784227 and proposed it to be a causal regulatory SNP targeting the gene *TOX3*. Furthermore, the risk allele rs4784227-T was shown to increase the binding affinity of the pioneer factor FOXA1, resulting in a fivefold decrease in *TOX3* gene expression. Here, we sought to verify the reported mechanism at the rs4784227 locus using the results from our database. Figure 4A shows a snapshot of the genomic region around rs4784227 from the embedded genome browser. The MCF-7 DNase tracks in Figure 4A clearly indicate that the GWAS SNP is located within open chromatin region. Furthermore, the “ReMap 2018 Peaks” track, which represent TF binding peak locations collected from ENCODE and Gene Expression Omnibus (GEO) datasets (Barrett et al., 2013; Cheneby et al., 2018), showed several TF binding sites, supporting that this SNP is likely a causal SNP. The eQTL results showed a negative correlation between the risk allele rs4784227-T and the mRNA level of *TOX3* in TCGA breast cancer samples (Figure 4B). Our motif analysis results further suggested FOXJ3 as one of the top candidate TFs (Figure 4C); given the similarity of FOXJ3 and FOXA1 motifs (*q*-value = 0.0098), as predicted by the Tomtom motif comparison tool from MEME web resource (Gupta et al., 2007; Bailey et al., 2009), our overall results were thus consistent with the findings of Cowper-Sal lari et al. (2012).

### Case Study (Novel): (rs1250003, *ZMIZ1*, GATA)

**FIGURE 5:**
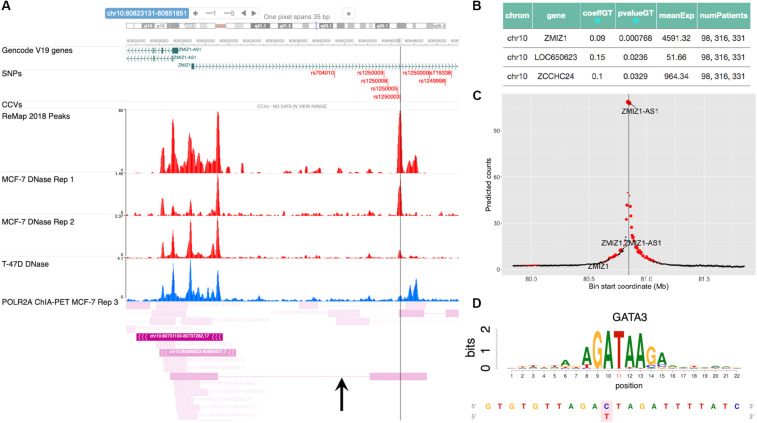
Case study of GWAS locus at *ZMIZ1*: (rs1250003, *ZMIZ1*, GATA). **(A)** Embedded genome browser showing the LD SNP rs1250003 within an open chromatin region (MCF-7 DNase track) and several TF peaks (“ReMap 2018 Peaks”). Black arrow indicates a chromatin looping interaction between the SNP locus and the *ZMIZ1* promoter. **(B)** eQTL results showing the top significant target gene of the GWAS SNP to be *ZMIZ1*. **(C)** Average predicted chromatin contact counts across several HiC-Reg models in MCF-7, showing the *ZMIZ1* promoter as one of the loci significantly interacting with the LD SNP. **(D)** Predicted TF candidate GATA motif from Kheradpour and Kellis (2014).

The SNP rs704010, residing within an intron of the gene *ZMIZ1*, was reported to be associated with increased breast cancer risk in Turnbull et al. (2010), and this association was subsequently verified in later studies (Michailidou et al., 2013, 2015, 2017; Zhang et al., 2018a). Figure 5A shows a snapshot of the locus from the embedded genome browser. Among the 12 high LD SNPs shown in the first track, we identified rs1250003 to be the only SNP residing within an open chromatin region in MCF-7 and also to a lesser extent in T-47D, as shown by the DNase tracks. This candidate SNP rs1250003 was located about 5 kb from the GWAS SNP and in high LD with the GWAS SNP (*r*^2^ = 0.99, 1000 Genomes Phase 3, EUR population). We also found that in the European population (1000 Genomes, Phase 3), rs1250003 was in perfect LD with two SNPs (rs1250008, rs1250009) previously reported to be CCVs (Fachal et al., 2020). Several TFs relevant to breast cancer – such as ESR1, FOXA1, and GATA3 – were found to bind near the SNP, as shown by the corresponding ChIP-seq tracks, indicating an important regulatory role of the SNP. The genotype status of rs704010 significantly correlated with the mRNA level of *ZMIZ1* (*p* = 7.7 × 10^–4^) (Figure 5B). POLR2A ChIA-PET track in MCF-7 further showed a chromatin-looping interaction between the SNP location and the promoter of *ZMIZ1* (Figure 5A). A significant interaction was also computationally predicted between the two loci in MCF-7 (Figure 5C). Our integrative analysis thus implicated *ZMIZ1* to be the top candidate target gene for the locus. Finally, we found GATA family binding motifs to be significantly disrupted by the SNP (Figure 5D), consistent with the ChIP-seq data. Thus, a quick analysis based on ABC-GWAS found the triplet (rs1250003, *ZMIZ1*, GATA) to be a novel putative functional mechanism behind the GWAS SNP rs704010 for increasing risk for breast cancer.

## Discussion

We demonstrated the capability of ABC-GWAS to find known, as well as novel, functional mechanisms of breast cancer GWAS loci. The computational and organizational framework of ABC-GWAS can be readily extended to other cancers. Once a (SNP, target gene, TF) triplet is identified through ABC-GWAS, several molecular experiments can be performed to validate the prediction. For example, the genotype of the predicted causative SNP could be modified through CRISPR-Cas9 base editors to study its effect on target gene expression (Komor et al., 2016). ChIP-quantitative polymerase chain reaction (ChIP-qPCR) is one way to measure how the SNP’s genotype status modulates the binding affinity of the predicted TF. ABC-GWAS thus provides a valuable resource, currently not available in other databases, for functional characterization of GWAS results. ABC-GWAS currently contains analysis results for only a predetermined set of SNPs, and a useful future extension could allow our integrative analysis pipeline to be performed on any genetic variant of interest chosen by the user. Another informative feature could be to provide a pathway analysis of candidate target genes and transcription factors in the context of breast cancer biology.

ABC-GWAS is an interactive web resource containing results from an integrative functional analysis of ER+ breast cancer variants. We combined data from TCGA, ENCODE, and several motif databases to create a comprehensive resource that includes an embedded genome browser with relevant tracks in breast cancer cell lines and several modules describing results from eQTL, motif, and expression correlation analyses. Using our resource, we have verified the known functional mechanism of a genetic variant regulating the gene *TOX3* and also proposed a novel mechanism targeting the *ZMIZ1* locus. ABC-GWAS aims to take GWAS discoveries to the next level by providing a one-stop resource for in-depth functional analyses critical for interpreting and prioritizing GWAS variants. We thus hope that our resource will help both experimental and computational researchers accelerate breast cancer research.

## Software Availability

1. Project name: ABC-GWAS.
2. Project home page: http://education.knoweng.org/abc-gwas/.
3. Operating system(s): Platform independent.
4. Programming language: HTML, JavaScript, R, Python.
5. Other requirements: JavaScript supporting web browser.
6. License: GNU GPL v3.0.

## Data Availability Statement

The datasets analyzed in the current study are available in the ENCODE project (https://www.encodeproject.org/) and the TCGA repository (http://cancergenome.nih.gov/) through GDC (https://portal.gdc.cancer.gov/projects) and dbGaP (https://www.ncbi.nlm.nih.gov/gap).

## Ethics Statement

The usage of NIH controlled-access datasets was approved by the NCBI dbGaP.

## Author Contributions

JS contributed to the conception and design of the project. MM, YZ, and SZ contributed to the data curation and analysis. MM, YZ, SZ, SR, and JS contributed to the methodology. MM and YZ developed the resource and visualization tools. SR, PP-P, and JS supervised the research and secured the funding. MM and JS wrote the original draft. All authors contributed to the article and approved the submitted version.

## Conflict of Interest

The authors declare that the research was conducted in the absence of any commercial or financial relationships that could be construed as a potential conflict of interest.
