# Supplementary material for: ABC-GWAS: Functional Annotation of Estrogen Receptor-Positive Breast Cancer Genetic Variants
Source: Front Genet. 2020 Jul 20;11:730. doi: 10.3389/fgene.2020.00730 (PMC7379852; doi:10.3389/fgene.2020.00730)
Supplement: Supplementary file 1 [file Data_Sheet_1.PDF]

Supplementary Material

Predicting SNP-promoter interactions in MCF-7 using HiC-Reg

Our SNP set comprised 70 GWAS SNPs and SNPs in high LD (r^2>=0.8) with the GWAS SNPs. We aimed to predict the long-range interactions for these SNPs in the MCF-7 cell line using HiC-Reg models trained in five cell lines with high resolution Hi-C data (Rao et al., 2014). In addition, we trained an additional model at 5 kb resolution using a sparse Hi-C dataset from MCF-7 (Barutcu et al., 2015). We processed the MCF-7 Hi-C dataset using HiC-Pro (Servant et al., 2015) into uniformly sized bins at 5 kb resolution and normalized these counts with ICE (Imakaev et al., 2012). For the five cell lines from Rao *et al.*, we downloaded the Hi-C SQRTVC normalized contact counts from GSE63525 (Rao et al., 2014).

We generated SNP-region interactions at the 5 kb resolution by binning the genome into 5 kb non-overlapping bins. We located the 5 kb bin for each LD and GWAS SNP (some bins have multiple SNPs) and generated SNP-bin pairs in the 1 Mb radius of the SNP bin. Each pair comprised one region containing the SNP and the other region within the 1Mb radius of the SNP region. We represented features of a region as a 14-dimensional feature vector, each dimension corresponding to one of the following 14 measurements extracted from 12 datasets from the ENCODE project for the six cell lines: GM12878, K562, HUVEC, NHEK, HMEC and MCF-7. These 12 datasets included 10 histone modifications ChIP-seq, CTCF ChIP-seq and DNase-seq. We used the following ten histone marks in the prediction models: repressive marks (H3K27me3, H3K9me3), marks associated with active gene bodies and elongation (H3K36me3, H4K20me1, H3K79me2), enhancer marks (H3K4me1, H3K27ac), and activating marks (H3K9ac, H3K4me2, H3K4me3). Two of the 14 features are DNase-seq derived motif instances of RAD21 and TBP, which we have seen as important contributors for predicting long-range interactions (Roy et al., 2015). A ChIP-seq signal for a region is represented as the average read count aggregated into a 5 kb non-overlapping region. For TBP and RAD21, we predicted the binding sites using PIQ (Sherwood et al., 2014) on the DNase I data from each cell line and used the sum of purity scores for all motifs mapped to the same 5 kb bin as the signal value. The purity score of a motif is proportional to its likelihood of being a true binding instance. To generate a feature vector for a pair of regions, we concatenated the 14-dimensional feature vectors of the two regions together with the feature vectors of the intervening region between the two regions, and genomic distance between the two regions to obtain a feature vector of 43 dimensions. We refer to this as the WINDOW feature following Whalen *et al.* (Whalen et al., 2016). The feature value of the intervening region is the mean signal value of the feature in all 5 kb bins in the intervening region.

To make predictions in the MCF-7 cell line, we trained 8 different models at 5 kb resolution: 3 Global Models (a MCF-7 CV model, a GM12878 HiC-Reg model, a HiC-Reg Ensemble model) and 5 Local Models. We trained an MCF-7 model using MCF-7 Hi-C data from Barutcu *et al.* (Barutcu et al., 2015). The GM12878 HiC-Reg model was trained on the entire chromosomes of GM12878 Hi-C data from Rao *et al.* (Rao et al., 2014). The HiC-Reg Ensemble model used the average of predictions from the global models trained on each of the five cell lines from Rao *et al.* (Rao et al., 2014). For the Local

Models, we transformed WINDOW feature to z-scores and trained on a 2 Mb block centered around the SNPs. Each local model was trained on one of the five Rao *et al.* (Rao et al., 2014) cell lines.

We applied all 8 models to predict contact counts between pairs of regions in the MCF-7 cell line. We next used a method based on a Binomial test from Duan *et al.* (Duan et al., 2010) to identify significant interactions for these SNP associated interactions (q-value < 0.05). For the 3 Global Models, significance testing was performed for all pairs per chromosome. For the Local Models, significance testing was performed for all SNP-region pairs associated with each SNP. For the significant interactions associated with each SNP, we mapped the interacting regions to nearest genes based on the overlap of a gene's TSS with the region. Specifically, we mapped a 5 kb region to a gene if there is overlap between the ± 1kb window around the TSS and the 5 kb region. If a gene had multiple TSS that overlapped with the interacting regions, we associated all of them to the SNP.

Supplementary Table 1. Source and accession/URL for the preloaded tracks in the Genome Browser.

Track name	Source	Accession/URL
Gencode v19 genes	GENCODE	https://egg.wustl.edu/d/hg19/gencodeV19.gz
ReMap 2018 peaks	ReMap	http://tagc.univ-mrs.fr/remap/index.php
MCF-7 DNase	ENCODE	ENCFF122HSD, ENCFF216EVD
T-47D DNase	ENCODE	ENCFF342HXQ
POLR2A ChIA-PET MCF-7	ENCODE	https://egg.wustl.edu/d/hg19/wgEncodeGisChiaPetMcf7Pol2InteractionsRep3.gz, https://egg.wustl.edu/d/hg19/wgEncodeGisChiaPetMcf7Pol2InteractionsRep4.gz
CTCF ChIA-PET MCF-7	ENCODE	https://egg.wustl.edu/d/hg19/wgEncodeGisChiaPetMcf7CtcfInteractionsRep1.gz, https://egg.wustl.edu/d/hg19/wgEncodeGisChiaPetMcf7CtcfInteractionsRep2.gz
ERa ChIA-PET MCF-7	ENCODE	https://egg.wustl.edu/d/hg19/wgEncodeGisChiaPetMcf7EraaInteractionsRep1.gz https://egg.wustl.edu/d/hg19/wgEncodeGisChiaPetMcf7EraaInteractionsRep2.gz, https://egg.wustl.edu/d/hg19/wgEncodeGisChiaPetMcf7EraaInteractionsRep3.gz
FOXA1 MCF-7	ENCODE	ENCFF561UXI
FOXA1 T-47D	ENCODE	ENCFF275YIF
GATA3 MCF-7	ENCODE	ENCFF145WHY
GATA3 T-47D	ENCODE	ENCFF129RJN
ESR1 T-47D	ENCODE	ENCFF234TXK, ENCFF655SMZ

Supplementary References

Barutcu, A.R., Lajoie, B.R., McCord, R.P., Tye, C.E., Hong, D., Messier, T.L., et al. (2015). Chromatin interaction analysis reveals changes in small chromosome and telomere clustering between epithelial and breast cancer cells. *Genome Biol* 16**,** 214. doi: 10.1186/s13059-015-0768-0.

Duan, Z., Andronescu, M., Schutz, K., McIlwain, S., Kim, Y.J., Lee, C., et al. (2010). A three-dimensional model of the yeast genome. *Nature* 465(7296)**,** 363-367. doi: 10.1038/nature08973.

Imakaev, M., Fudenberg, G., McCord, R.P., Naumova, N., Goloborodko, A., Lajoie, B.R., et al. (2012). Iterative correction of Hi-C data reveals hallmarks of chromosome organization. *Nat Methods* 9(10)**,** 999-1003. doi: 10.1038/nmeth.2148.

Rao, S.S., Huntley, M.H., Durand, N.C., Stamenova, E.K., Bochkov, I.D., Robinson, J.T., et al. (2014). A 3D map of the human genome at kilobase resolution reveals principles of chromatin looping. *Cell* 159(7)**,** 1665-1680. doi: 10.1016/j.cell.2014.11.021.

Roy, S., Siahpirani, A.F., Chasman, D., Knaack, S., Ay, F., Stewart, R., et al. (2015). A predictive modeling approach for cell line-specific long-range regulatory interactions. *Nucleic Acids Res* 43(18)**,** 8694-8712. doi: 10.1093/nar/gkv865.

Servant, N., Varoquaux, N., Lajoie, B.R., Viara, E., Chen, C.J., Vert, J.P., et al. (2015). HiC-Pro: an optimized and flexible pipeline for Hi-C data processing. *Genome Biol* 16**,** 259. doi: 10.1186/s13059-015-0831-x.

Sherwood, R.I., Hashimoto, T., O'Donnell, C.W., Lewis, S., Barkal, A.A., van Hoff, J.P., et al. (2014). Discovery of directional and nondirectional pioneer transcription factors by modeling DNase profile magnitude and shape. *Nat Biotechnol* 32(2)**,** 171-178. doi: 10.1038/nbt.2798.

Whalen, S., Truty, R.M., and Pollard, K.S. (2016). Enhancer-promoter interactions are encoded by complex genomic signatures on looping chromatin. *Nat Genet* 48(5)**,** 488-496. doi: 10.1038/ng.3539.
